# A Growing Troubling Triad: Diabetes, Aging, and Falls

**DOI:** 10.1155/2013/342650

**Published:** 2013-02-16

**Authors:** Ryan T. Crews, Sai V. Yalla, Adam E. Fleischer, Stephanie C. Wu

**Affiliations:** Center for Lower Extremity Ambulatory Research (CLEAR), Dr. William M. Scholl College of Podiatric Medicine, Rosalind Franklin University of Medicine and Science, 3333 Green Bay Road, North Chicago, IL 60064, USA

## Abstract

There is a significant and troubling link between diabetes (DM) and falls in the elderly. Individuals with DM are prone to fall for reasons such as decreased sensorimotor function, musculoskeletal/neuromuscular deficits, foot and body pain, pharmacological complications, and specialty (offloading) footwear devices. Additionally, there is some concern that DM patients are prone to have more severe problems with falls than non-DM individuals. Fractures, poorer rehabilitation, and increased number of falls are all concerns. Fortunately, efforts to mitigate falls by DM patients show promise. A number of studies have shown that balance, strength, and gait training may be utilized to successfully reduce fall risk in this population. Furthermore, new technologies such as virtual reality proprioceptive training may be able to provide this reduced risk within a safe training environment.

## 1. Introduction

From 2000 to 2010 the elderly (65+ years) population in the USA has continued its upward trend, increasing by 5.25 million (15%) to a total of 40.26 million [1]. This amounts to 13% of the entire population [1]. Thanks to the aging baby boomers population, by the year 2050 the elderly population is expected to reach 88.5 million, which would represent 20% of the total population [2]. One of the greatest health challenges facing this population is falls. In 2000 there were a reported 10,300 fatal falls by the elderly in the USA that incurred $179 million in direct medical costs [3]. There were an additional 2.6 million medically treated falls that cost $19 billion in medical costs. Other western nations report similar significant burdens with the United Kingdom reporting *£*981 million (US $1.9 billion) in costs for falls in those 60 or more years old in 1999 [4], and in 2001 the annual cost of care attributable to falls in those 65 or older in Australia was $86.4 million (US $66.1 million) [5]. While the cause of falls is often multifactorial, diabetes mellitus (DM) has been shown to be a significant factor. The significance of the relationship between aging, DM, and falls has been highlighted by previous work that found the annual incidence of falls in elderly individuals with DM to be 39% [6]. This paper will review the association of fall occurrence and diabetes, the association of fall severity and diabetes, and efforts to limit diabetes associated risks for falls.

## 2. Association between Diabetes and Falls

Falls are a major concern for elderly adults with DM [7]. The high prevalence of falls in ambulatory elderly individuals with DM is well established with reported annual incidence rates of 39% in those over 65 years [6] and 35% in those over 55 years [8]. In addition to the reported high incidence of falls in this population, it has been established that DM individuals are at a higher risk for falls [9, 10]. There are a number of mechanisms by which DM may contribute to falls. Decreased sensorimotor function, musculoskeletal/neuromuscular deficits, foot and body pain, pharmacological complications, and specialty (offloading) footwear devices will be discussed.

## 3. Decreased Sensorimotor Function

Diabetic peripheral neuropathy (DPN) is common among the DM population, and its prevalence increases with age and duration of diabetes [11–13]. While a number of detrimental changes to the nervous system fall under the umbrella of DPN, this section will focus on the most common type which is damage to the large nerve fibers that results in decreased sensorimotor function [14]. DPN patients with diminished plantar sensation on their feet have been observed to exhibit increased postural sway along with significant loss of postural control [15, 16]. Loss of proprioceptive feedback [17] during standing and walking in turn leads to increased risk of falls which is evident from a recent prospective cohort study on 9,249 women aged above 67 years where postural instability and DPN were observed to account for the largest percentage of the relationship between diabetes and falls [18]. Cross-sectional studies have also found a strong association between the development of DPN and falls. Among 21 DM patients over 55 years who reported at least one fall in the past year, MacGilchrist et al. found that 86% had peripheral neuropathy [8]. Furthermore, it has been shown that as DPN severity increases, performance on functional reach tests declines [19]. Thus, as DPN severity increases, there is a higher risk of falls occurring while completing reaching tasks in the standing position. While there are many risk factors that contribute to falls, DPN is definitely a significant contributor [20].

## 4. Musculoskeletal/Neuromuscular

Apart from DPN, lower physical activity, muscle strength, and poor postural control were also found to be among the significant risk factors that influence gait patterns and increased risk of falls among the DM population [21, 22]. Among the elderly population, postural control is an important factor to perform activities such as standing, sitting, walking, and reaching tasks [23–25]. Considering that the feet serve as the base supporting structure during these activities, the strength in lower extremity joints plays a vital role in establishing a strategy for postural stability [26]. Impaired postural control during static balance tests [27] as well as dynamic short whole body anterior translations of 1–4 mm in older patients with DM [28] increases the limitations at the base of support and in turn results in increased risk of falls.

In addition to the lack of sensorimotor function discussed previously, coordination of muscles for postural compensatory strategies is challenged in individuals with DPN. Najafi et al. [17] utilized a novel compensatory index for quantifying postural control strategy to compare strategies utilized by healthy young subjects to strategies of older DPN subjects. In comparison to the healthy young, the older DPN subjects had a significant 10% reduction in postural compensatory strategy. This was coupled with a 98% increase in postural sway. This difficulty in postural control coupled with an altered gait pattern [29] further increases the risk of falls in DPN patients.

Low plantar flexion strength has also been observed to be associated with increased center of mass (COM) displacement or sway among DM patients negatively affecting the maximum forward reach distance [24]. Accordingly, while studying ambulatory DM patients, Macgilchrit et al. found that ankle plantar flexion muscle strength was lower among fallers by 40% compared to nonfallers [8]. Reduced muscle strength has also been shown to result in reduced walking speeds [25, 30], and an increased double support phase of the gait cycle. Studies have shown increased double support time to be a significant factor for falls [31–33] especially in people with postural instability [34]. Individuals at high risk for falls likely adopt this increased double support strategy in order to limit the time during which they must maintain balance on a single limb. This further emphasizes the need for exercise training and developing a stable postural control strategy in DM patients to reduce the risk of falls.

## 5. Foot and Body Pain/Pharmacological Complications 

While the majority of excess fall risk in patients with diabetes can be attributed to DPN sensorimotor decrements and aberrant neuromuscular control, it is important to recognize that other factors associated with diabetes (e.g., foot and body pain and the use of psychotropic medications and polypharmacy) can also contribute to a heightened fall risk profile.

Foot pain is another recognized risk factor for falls among community-dwelling older adults [35, 36]. Patients with diabetes frequently experience symptoms of painful polyneuropathy as the distal nerve fibers in the toes and foot begin to deteriorate. Similarly, patients with chronic disabilities encounter greater levels of chronic, generalized body pain [37, 38] which also places them at increased risk for recurrent falls [21]. This is because diabetic individuals suffering with chronic pain may be less capable of adhering to productive self-management practices like regular exercise [37] and have poorer mental health and physical functioning [38] which places them at increased risk for falls [35]. 

Diabetic patients that suffer with neuropathic pain are frequently managed with psychotropic and other central nervous system mediated medications. Amitriptyline and duloxetine hydrochloride, for example, are commonly used to treat the painful symptoms of diabetic neuropathy, the latter being one of only two FDA-approved medications for use in diabetic neuropathy. Psychotropic medications are frequently implicated in falls and nearly double an elderly adult's risk for experiencing a fall [39, 40] and having recurrent falls [21, 41]. Older adults suffering with diabetes are also more likely to be taking a greater number of prescription medications [21] and seem to be more sensitive to the effects of polypharmacy than their nondiabetic counterparts [40, 42]. Patients with diabetes start to experience an increased risk of falling with regimens involving just 4 or more prescription medications [42]. 

One of the hazards of managing diabetes is the increased risk for experiencing unexpectedly low blood glucose levels and symptomatic hypoglycemia. Hypoglycemic episodes can occur with oral hypoglycemic and/or insulin use and frequently result in a state of dizziness, confusion, and postural instability which increases ones' risk for a fall accident [43–46]. While the literature has been somewhat mixed regarding the extent to which the level of diabetes control influences fall risk [42, 47, 48], it remains clear that the medications associated with treating DM and its complications can contribute to increased fall risk. 

## 6. Offloading Footwear

Footwear such as athletic shoes has been found to reduce fall risk in older adults [49, 50]. Within the DM population, foot ulcers are highly prevalent [51, 52] and often develop due to cumulatively high localized plantar pressure on their feet [53–55]. In order to reduce the risk of ulceration and also for treatment of ulcers, footwear that provides offloading of the localized stress is widely used [55–57]. Even though offloading footwear has not been directly associated with falls, some offloading devices have certainly been found to negatively affect postural stability [58, 59]. Of most concern are the casts and cast walkers used in the treatment of diabetic foot ulcers. These devices significantly restrict normal gait. In addition to being heavy, prohibiting ankle movement, prohibiting normal heel to toe progressive loading of the foot, and potentially decreasing proprioception, some offloading footwear also creates a limb length discrepancy [60]. Given the association of postural stability to fall risk [61], reduced postural stability due to offloading footwear will increase the risk of falls of those utilizing the footwear. Design modification for offloading footwear such as reduction in strut height and reduced weight has been suggested as a means to improve postural stability [62] which might in turn reduce fall risk.

## 7. Association of Fall Severity and Diabetes

In an editorial concerning complications of diabetes in elderly people, Gregg et al. noted that falls and fractures along with cognitive disorders, physical disability, and other geriatric syndromes may be as great a concern to older people with diabetes as the more traditionally recognized vascular complications [63]. Diabetes increases not only the risk of falls, but also the risk of fractures [64, 65]. Strotmeyer and coworkers found older adults with diabetes to be at higher fracture risk compared with nondiabetic adults with similar bone mineral density [65]. The literature suggests that the fracture risk point estimates described in type 1 diabetes are considerably higher than in type 2 diabetes [66]. However, increased fracture risk in longstanding type 2 diabetes is a paradoxical phenomenon because men and women with type 2 diabetes typically have normal to high bone mineral density [67–69]. Altered body composition and microvascular complications, including retinopathy, peripheral and autonomic neuropathy, hypoglycemia, and use of medications, particularly thiazolidinediones, are all related with increased risk of fractures in older adults with diabetes [7, 70].

In addition to a predisposition to fractures with falls, individuals with DM may be prone to poorer rehabilitation. In investigating rehabilitation following hip fracture, Semel et al. [71] found that patients with diabetes had worse outcomes. The authors noted that patients with diabetes had a worse length of stay efficiency (a measure of recovery per each day of hospital stay) compared with other patients. Similarly, when Liberman et al. compared 224 patients with diabetes to 738 patients without diabetes in a prospective cohort study, they found that patients with diabetes had a worse functional outcome following rehabilitation after hip fracture surgery [72]. Ekstrom and colleagues evaluated the health-related quality of life (HRQoL) after hip fractures and noted that patients with diabetes mellitus had more pain, comorbidities, and reduced health status preoperatively than patients without diabetes. The authors further noted that while there were no more medical complications among patients with diabetes during the first postoperative year, cardiac (*P* = 0.023) and renal failures (*P* = 0.032) were more frequent in patients with diabetes at 24 months.

One last factor to consider in the severity of falls is the occurrence of recurrent falls. Pijpers et al. compared the incidence of recurrent falls in older people with and without diabetes with a mean followup of 139 weeks and noted that 30.6% of the individuals with diabetes and 19.4% of the individuals without diabetes fell recurrently (incidence rate of 129.7 versus 77.4 per 1,000 persons-years, respectively, HR = 1.67 (95% CI: 1.11–2.51)) [21]. The authors noted that the greater number of medication, higher levels of pain, poorer self-perceived health, lower physical activity and grip strength, more limitations in activities of daily living, lower-extremity physical performance, and cognitive impairment may potentially increase the risk of recurrent falls, and these variables together accounted for 47% of the increased risk of recurrent falls associated with diabetes (adjusted HR = 1.30 (0.79–2.11)).

## 8. Combating DM-Related Fall Risks

A recent publication regarding falls of elderly people in long-term care facilities found that 49% of falls in this setting occurred while walking, 24% while standing, and 21% while either rising up or lowering oneself [73]. In studying daily physical activity patterns of DPN subjects with a mean age of 59 ± 8 years, it was found that each 24 hr day these subjects spend 13.5% of their time standing and 6.1% walking and performed 77 sit-to-stand postural transitions per day on average [74]. Therefore, unfortunately there are plenty of opportunities for elderly adults with DM to experience a fall. Accordingly, numerous investigations regarding improving balance, strength, and gait in order to reduce falls have been conducted [27, 30, 75–77]. 

Weekly balance training sessions with or without additional strength and/or gait training have been shown to reduce fall risk in DM patients [27, 30, 75, 76]. Positive outcomes have been found both in the broad perspective of DM patients in general [76] as well as specifically in DPN patients [30, 75]. What is more promising is that in a study comparing four groups (DM with fall history *n* = 7, DM without fall history *n* = 9, non-DM with fall history *n* = 7, non-DM without fall history *n* = 14), the greatest improvements were seen in DM patients with a history of falling [27]. One study to actually track fall occurrence following implementation of a strength and balance program for DPN patients did not show a reduction in falls compared to a DPN control group; however, there were several limitations to the study [77]. This was a secondary analysis of a study utilizing subjects with a somewhat low minimum age criteria of 50. Also the majority of the prescribed intervention was to be conducted at home without supervision. Only 8 training sessions were conducted with a physical therapist, all of which occurred during months 1–3. The first balance and strength assessments to occur after initiation of training did not occur until 6 months after study initiation. Finally no information was provided concerning compliance of exercise at home and only 45% of participants in the intervention group completed “more than half of the required study protocol elements (p. 1572).” In contrast, a study comparing home-based versus “center-based” balance and strength training for 107 community-dwelling adults (DM was not an inclusion criteria) referred for a falls prevention service found that the center-based service demonstrated significantly better results in preventing falls [78]. 

In addition to traditional balance and strength training, new technologies utilizing virtual reality may provide additional training methods with limited patient risk. It has been previously shown that DM patients either without or with minimum DPN demonstrate reduced toe-obstacle clearance with altered gait patterns during obstacle crossing [79]. In addition to increasing fall risk during daily living, training to improve clearance could be risky in that falls may occur during training sessions requiring subjects to step over obstacles. Recently an investigation validated a virtual reality protocol for assessing obstacle crossing while stepping in place [80]. The study showed that DPN participants had greater difficulty completing the virtual obstacle crossing. It is possible that this paradigm could be used as a minimal risk training program to improve real world obstacle crossing and subsequently reduce fall risk. 

## 9. Conclusion

Falls in elderly individuals with DM are a significant burden to the healthcare system. A number of factors tied to DM predispose this population to a higher risk of falls. Additionally, the falls that this population suffers from have the potential to be more severe in terms of injuries sustained as well as the recovery process. Therefore much work is ongoing regarding the reduction of falls in this population. Numerous studies utilizing balance, strength, and/or gait training have demonstrated reduced fall risk for DM patients that undertook the training. More prospective work is needed regarding the long-term outcome of these interventions on actual fall prevention.
